# A New Food Composition Database of Lactose-Free Products Commercialized in Spain: Differences in Nutritional Composition as Compared to Traditional Products

**DOI:** 10.3390/foods10040851

**Published:** 2021-04-14

**Authors:** María Martínez Rodríguez, Mᵃ de Lourdes Samaniego-Vaesken, Elena Alonso-Aperte

**Affiliations:** Research Groups C08/0720 Food and Nutrition in Health Promotion2 and E02/0720 Nutrition for Life3, Departamento de Ciencias Farmacéuticas y de la Salud, Facultad de Farmacia, Universidad San Pablo-CEU, CEU Universities, Urbanización Montepríncipe, 28925 Alcorcón, Spain; mar.martinez1.ce@ceindo.ceu.es (M.M.R.); l.samaniego@ceu.es (M.d.L.S.-V.)

**Keywords:** lactose-free, dairy products, market study, food composition database, label information

## Abstract

We developed a new database to evaluate the nutritional composition of lactose-free products from Spain. The database includes dairy products and other products, all of which show the “lactose-free” declaration on their label, accounting for 327 products in total. Of these, 123 are dairy products, 16 are non-dairy products which include a dairy ingredient (5%) and 188 items (57% of the sample) are non-dairy products that do not contain any dairy ingredient. The main subgroups are yogurt (25%), milk (24%), and cheese (17%). Nineteen percent of the compiled products included nutritional claims on their labels. Most lactose-free products did not contain either added sugars or low- or no-calorie sweeteners (58%), while 34% included added sugars and only 6%, sweeteners or a combination of both (2%). We found that 19.5%, mainly within the milk subgroup, were fortified with vitamins A, D, E, K, B_9_, and B_12_, P, and Ca. There were no significant differences in the nutritional composition between lactose-free products and traditional products. According to the NOVA classification, 55% of compiled lactose-free products were ultra-processed, and 20% processed. The array of lactose-free products marketed in Spain proves that there are enough, both in quantity and quality, to satisfy the dairy needs of lactose intolerants.

## 1. Introduction

Lactose (4-O-β-d-galactopyranosyl-d-glucose) is the unique sugar disaccharide present in the milk of most mammals at concentrations from 4.5 to 5.0 g/100 mL [1]. Mammals need to digest lactose, so there is a lactase enzyme in the microvillus membrane of the enterocytes in the small intestine, which hydrolyzes lactose into its monosaccharide units, galactose and glucose, which are then further absorbed [2]. Lactose intolerance occurs when the body is unable to produce the necessary amount of lactase and, consequently, undigested dietary lactose enters the large intestine, where it acts as a fermentable substrate for the colonic microflora [1]. Lactose intolerance comprises osmotic diarrhea and the symptoms of discomfort produced by lactose fermentation, i.e., bloating, flatulence, and cramps.

The impaired ability to digest lactose varies widely per country and per continent, from 98–100% of adults in Southeast Asia to just 1% in the Netherlands [3]. Less than 15% of the Northern European population is lactose intolerant, while 15–30% of the citizens in Central Europe suffer from it. This incidence increases up to 60% in the Mediterranean countries [4]. A recent review by Anguita-Ruiz et al. [5] gathers all lactose persistence phenotype and genotype frequency data reported worldwide. According to this source, only one-third of humans can digest milk and other lactose-rich dairy products during adulthood. In Spain, frequencies of lactase persistence range from 47% to 91%, depending on the populations studied [5].

Lactose intolerance is not a new topic, since the genetic analysis of remains from the first civilizations in the Neolithic period (8000 B.C.E.) proves that our ancestors suffered from primary lactase deficiency. Moreover, in 400 B.C.E., Hippocrates described a series of intestinal symptoms in some people after consuming dairy products. Nonetheless, it was not until 1950, when dairy product consumption started to expand on a global scale, that the first documented medical cases of lactose intolerance were published [4].

Diagnosed lactose-intolerant subjects need to eliminate lactose from their diet, i.e., all dairy and a wide range of processed foods, so a daily provision of lactose-free alternatives is imperative. Moreover, consumer research shows that other lactose-tolerant or undiagnosed population groups actively search for these products for several potential health-related issues or benefits. Consequently, the demand for lactose-free products on the market is rising. 

Lactose-free or lactose-hydrolyzed processed products are those that would contain a dairy product as the main ingredient (i.e., cheese and yogurt), or those that incorporate dairy ingredients in a minor proportion to provide a specific technological or sensory function within the product (i.e., dehydrated milk, whey concentrate, etc.). In either case, the Spanish Agency of Food Safety and Nutrition (AESAN) has established that a food product labeled as “lactose-free” should show an absence of lactose in accordance with the most sensitive analytical techniques, that is, less than 0.01% lactose [6]. Moreover, Regulation (EU) 1169/2011 of the European Parliament and of the Council of 25 October 2011, on the provision of food information to consumers [7], establishes that lactose is an ingredient that mandatorily must be declared in the ingredient lists of food products, as well as other information means (i.e., menus). These directions aid population groups, such as lactose intolerant individuals, to choose the most suitable food options. However, patients diagnosed as lactose intolerant must be advised to avoid risky foods that may be inadequately labeled, including processed meats, bread, cake mixes, sauces, soft drinks, and lagers [8]. There are other food products that are naturally lactose-free and are used as dairy or milk substitutes, such as beverages made of vegetable protein sources like oats or almonds, which also show the lactose-free claim on their labels. In the present study, we only assessed those foodstuffs that contain at least one dairy ingredient.

The enzyme used to produce lactose-free dairy products has traditionally been the neutral β-galactosidase derived from the dairy yeast *Kluyveromyces lactis* (and its close relatives *Saccharomyces lactis*, *K. marxianus* or *K. fragilis*). There are different processes used in the dairy industry to produce lactose-free products, but in most of them, lactase is added. Currently, two processes (batch and aseptic) are in use to produce lactose-free milk, and both these processes use soluble lactase enzymes [9]. All fermented milk products and other dairy products can also be made by using lactose-free milk or by adding the lactase enzyme, but most yogurt producers opt to treat with lactase in the process. In addition, many other dairy products, such as cheese, ice cream, and desserts, are made lactose-free using an enzymatic treatment [10]. Lactase addition in yogurts is, moreover, a means of reducing sugar content, since lactose hydrolysis enhances sweetness in the product [11,12]. 

In 2010, the Scientific Opinion published by the European Food Safety Authority (EFSA) stated that no negative nutritional consequences could be expected from the consumption of lactose hydrolyzed dairy products, in either lactose intolerants or healthy people, if the only difference between conventional and lactose-free dairy products was their lactose content. However, it was also noted that limiting or removing conventional dairy products from the daily diet, without supplementation or adaptation of dietary habits, may result in low intakes of calcium, vitamin D, and riboflavin [13]. For instance, when lactose intolerance has an early onset, it might even impair the achievement of an adequate peak bone mass and may, therefore, predispose sufferers to severe osteoporosis at later ages [1].

Regardless of the increased need to provide lactose-free alternatives, at present, there is no information on their availability and suitability for lactose intolerants, or their nutritional composition, since these products are not included in food composition databases (FCDBs). Food composition data are important for a wide range of stakeholders and users including researchers, public food and health policymakers, healthcare professionals, industry (food, agriculture, software developers), consumers, and for educational purposes [14]. Data are used to convert food into its basic components, enabling health professionals and researchers to calculate nutrient intake and assess diet quality. Moreover, FCDBs are frequently used by the food industry, policymakers, and researchers to regulate and innovate in food production, and to develop dietary monitoring programs. Finally, knowledge of food composition empowers consumers to make healthy food choices.

The aim of the present work is, therefore, to evaluate the availability and composition of lactose-free products in Spain, and to acquire sound data to be included in Spanish FCDBs.

## 2. Materials and Methods

### 2.1. Design and Data Collection

We conducted an observational study of a representative sample of lactose-free products commercialized in Spain. Because the nutritional composition of lactose-free products is unknown, since they are not included in food composition databases (FCDBs), we designed a new tool to compile and assess the nutrient composition of these products. The database included all products with a “lactose-free” declaration on their label.

The online e-commerce platforms of major supermarkets and food retailers were selected to perform queries and identify all potential lactose-free products. The main brands were selected according to a market study carried out by Kantar Worldpanel, which collected sales data from 2017 and was published in 2018 [10,11].

The field study was conducted in Madrid, between October 2018 and January 2019. It included manufacturers’ brands and own-label or distributor brands. According to available brand sales data [15], the study covers 80% of the market share. In addition, retailers were visited to complete the sampling of lactose-free products not previously identified online.

### 2.2. Food Database Development

Since this study was based on the information provided by the product’s label, photographs were taken to collect label data, including product name, weight, recommended serving, ingredients, macronutrient profile, nutrition claims, health claims, disease risk reduction claims, and other statements. Four to five photographs, including every side of the packaging, were taken from each product. The aim of these photographs was to accurately collect the nutritional data declared on the labels of lactose-free products. Data were collected in a fit-for-purpose designed FCDB created in Microsoft^®^ Access (Microsoft Corp., Washington, DC, USA). Products were classified according to the Langual^™^ [16] and the FoodEx2 [17] food group classification systems, which are widely used in European FCDBs.

### 2.3. Ingredients and Nutritional Information Study

To assess nutritional quality, we compiled the following information from the product label:

(a) Nutritional composition, including energy (kcal), protein (g), carbohydrates (g), sugar (g), fat (g), saturated fatty acids (g) and salt (mg) per 100 g of edible portion.
(b) Nutritional claims, health claims, and disease risk reduction claims.
(c) Added sugars and low- and no-calorie sweeteners (LNCS).
(d) Fortification, defined as the addition of extrinsic vitamins and/or minerals.

We relied on data declared by manufacturers, as we did not perform any chemical analysis of the proximate composition of products.

Data quality control to check for errors during and after completion of data input was assessed independently by two compilers, following recommendations from the EuroFIR guidance for data compilation [18]. Values were manually inspected and corrected for coherence throughout the entire FCDB, by identifying low or high outlying values, missing data, and zero values assigned by mistake. Duplicate products were removed.

To compare the nutritional composition, we selected matched lactose-containing products from commercial brands. Milk, yogurt, cheese, milkshakes and dairy beverages, butter and cream, and dairy desserts were selected for this comparison as they represented the main subgroups. Milk varieties (with and without lactose) included whole, semi-skimmed, skimmed, and calcium-enriched milk. In the case of yogurt (with and without lactose) varieties included for comparison were natural, sweetened, flavored, semi-skimmed, sugary, Greek, Bifidus, and liquid yogurt. In the case of cheese, we categorized products into fresh cheese and matured cheese. 

### 2.4. Statistical Analysis

Differences were assessed by statistical analysis, which was performed as a descriptive analysis of the sample with the main quantitative variables expressed through centralization and dispersion parameters. Results are reported as average ± standard deviation per group or as a percentage. The Kolmogorov–Smirnov test was used to analyze whether the samples were parametric or non-parametric. Non-parametric data were compared using the Mann–Whitney U, Wilcoxon or Kruskal–Wallis tests and when they resulted in differences, multiple comparisons between medians were studied by the t-student test, adjusting the p-value with the Bonferroni correction. Differences were considered significant at *p* < 0.05. Data analysis was completed with the SPSS 24.0 software package (IBM Corp., Armonk, NY, USA).

## 3. Results

**Figure 1:**
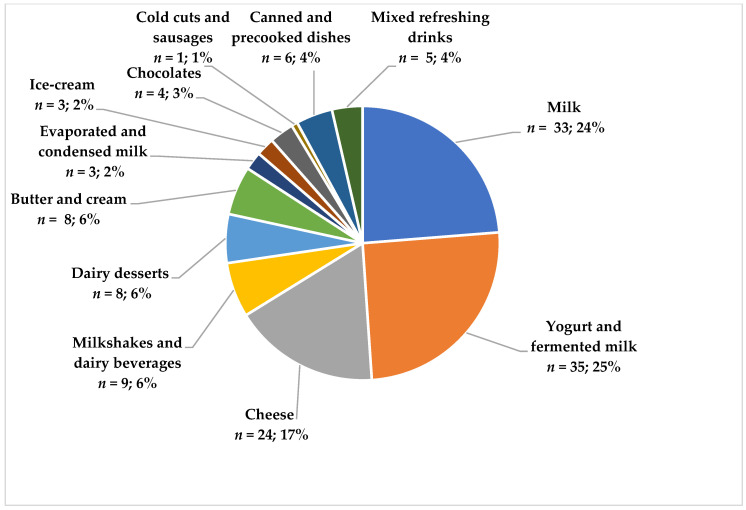
Types of lactose-free foods included in the food composition database (FCDB).

The lactose-free food composition database (FCDB) included 327 products that were declared to be lactose-free on their labels. From these, 123 (37.6%) were dairy products, 16 (4.9%) were non-dairy products that included a dairy ingredient, and 188 items (57.5% of the sample) were non-dairy products that did not contain any dairy ingredient (and therefore could not possibly include lactose). Those lactose-free labeled products that did not contain a dairy ingredient, or did not contain lactose as an additive, were excluded from further study. Lactose-free products comprised eleven subgroups, of which yogurts (*n* = 35), milk (*n* = 33), and cheese (*n* = 24) accounted for more than 70% of the total (Figure 1). We found that up to 63% of lactose-free products were marketed by manufacturers’ brands, while only 37% were marketed by distributor’s brands (white-label products). The database is available for research purposes on demand from the corresponding author.

It is remarkable that even though an important number of non-dairy products (57% of the sample) did not contain any dairy ingredient or lactose in their formulation, they still declared to be lactose-free on their labels. These products belonged to the following subgroups: pastries, bread, biscuits, puff pastry and pizza bases, cold cuts and sausages, canned and pre-cooked dishes, broths, and sauces.

The lactose-free FCDB included nutritional values, lists of ingredients, and nutritional and health claims when available. The nutritional composition of lactose-free dairy products was studied by food subgroup (Table 1). As expected, the total fat and saturated fat contents per 100 g were higher within the butter and cream subgroup as well as in the cheese subgroup, being both the most energy-dense subgroups. In turn, carbohydrate contents from evaporated and condensed milk, ice-cream and dairy desserts were higher, while the cheese subgroup provided a significant amount of protein. Serving sizes of each subgroup should be considered to assess the actual nutrient contribution of each group to dietary intakes.

The studied lactose-free dairy products showed a similar nutritional composition to traditional products, as there were no significant differences between them. Comparisons were made between lactose-free and traditional milk, yogurt, cheese, milkshakes and dairy beverages, butter and cream, and dairy desserts, as these were the major categories included in our FCDB (Table 2). In the case of milk, the varieties assessed were whole, semi-skimmed, skimmed, and calcium-enriched. The varieties of yogurts used for comparison were natural, sweetened, flavored, semi-skimmed, sweetened, Greek, Bifidus, and liquid. The different types of cheeses used were fresh cheese and matured cheese. As expected, there were no significant differences in protein contents between traditional and lactose-free milk. In addition, no significant differences were found in sugar contents between traditional and lactose-free yogurt (Table 2).

Nineteen percent of the collected products included nutritional claims on their labels (Table 3). Examples of these claims were “low-fat” and “no added sugar”, while the majority (81%) did not declare any. Only 2% of products presented health claims in accordance with Regulation (EC) No 1924/2006 [19] and Regulation (EU) No 432/2012 [20] such as “Calcium and vitamin D are necessary for bone growth and development”.

Most compiled lactose-free products did not contain either added sugars or low- or no-calorie sweeteners (LNCS) (58%), while 34% included added sugars and only 6%, LNCS or a combination of both (2%). Types of added sugars and LNCS ingredients are detailed in Table 4. Yogurts and smoothies were the groups that most frequently contained these ingredients, and the most used sweeteners were common sugar (sucrose), and acesulfame K plus sucralose.

When studying fortification frequency in lactose-free products, we found that 19.5% were fortified, specifically with vitamins A, D, E, B_9_, B_12_ and minerals Ca, K and P, and mainly within the milk subgroup (Table 5).

**Figure 2:**
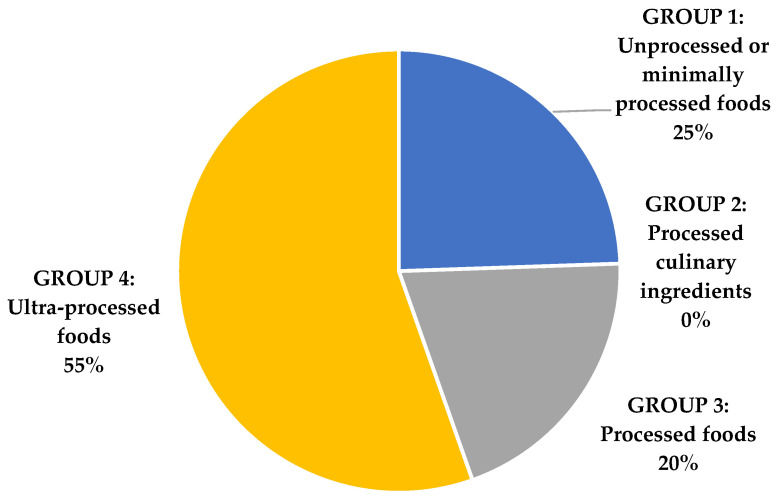
Degree of processing of lactose-free products as categorized by the NOVA classification.

According to the NOVA classification [21], 55% of compiled lactose-free products were ultra-processed, and 20% processed (Figure 2). The ultra-processed group included yogurt and fermented milks with added sugars, dairy desserts, milkshakes, ice-cream, chocolates, juices, preserves, prepared meals, and cold cuts. According to NOVA [21], yogurt that contains added sugar is considered an ultra-processed product, while natural yogurt is not.

Most milks, yogurts, creams, and butters declared the presence of lactase as an ingredient (Table 6). β-Galactosidases, including lactase, are employed by many food industries to degrade lactose and improve the digestibility, sweetness, solubility, and flavor of dairy products [12]. In accordance with European labeling regulations, lactase addition must be included in the ingredients list [7]. Moreover, all products in the evaporated and condensed milks group contained lactase. However, none of the ice cream, chocolate, cold cuts, preserves and precooked foods, and juices declared lactase as an ingredient. For the rest, in the same subgroup, some products declared lactase and others did not.

## 4. Discussion

The first lactose-free milk was launched on the market in Spain in 2006 [22]. Since then, an increasing number of products have been developed to satisfy the high demand for lactose-free products. The present work encompasses the compilation of a representative sample of lactose-free foods that are currently available to and consumed by the Spanish population. It is the first one of its kind, and it includes 327 products from 43 different brands. Furthermore, this study compares the differences between the nutritional composition of lactose-free products with their counterpart lactose-containing products. Results showed that there is no difference between lactose-free products and lactose-containing products for any specific nutrient.

Product labels are the most readily available way to study nutritional composition, as analytical values are limited by the expensiveness of compiling a representative sample from the market. For this reason, our lactose-free FCDB was built using the information and nutritional content provided by the label. Other studies like the ones from Carter et al. [23] and Webster et al. [24] also showed the advantages of using this approach, and we have also used it previously to build a gluten-free FCDB [25]. 

The presence of the lactose-free symbol or logo is widespread. Lactose is a common commercial food additive in non-dairy products, frequently used by the food industry due to its low price, texture, flavor, and adhesive qualities [3]. Therefore, it can be present in foods such as processed meats (sausages/hot dogs, sliced meats, pâtés), margarines, sliced bread, breakfast cereals, potato chips, processed foods, ready-to-eat meals, sauces, meal replacements (powders and bars), protein supplements (powders and bars), and even in beer of the milk stout style, and drugs. Consequently, it comes as no surprise that the market is flooded with products bearing the lactose-free logo. In addition, food ingredients commonly found, including amongst others lactoserum, whey, milk solids, and modified milk ingredients, should be avoided by lactose-intolerant subjects, since these products might contain lactose [3]. In these cases, the lactose-free logo would be totally justified. Remarkably, most lactose-free products (57%) identified in our market study did not contain any ingredient justifying this claim. 

Food labeling represents a powerful tool for consumers. According to the latest monographic study of the Spanish Ministry of Agriculture, Fisheries and Food, 7 out of 10 individuals declare they always read the label before buying a product [26]. Above all, they check for information regarding the expiration or preferred consumption date and the list of ingredients, as well as the preservation conditions and use of the product. Nutritional information is also essential for consumers, since 87.6% of those interviewed consider it important and useful, fats, sugars, and calories being the nutrients they pay the most attention to. Such information can provide the consumer with a clearer idea of the suitability of a particular product for his or her dietary needs [3].

The lack of standardization amongst lactose-free labeling declarations, observed by the presence of non-regulated or standardized symbols, could lead to consumer misunderstanding. It is difficult to identify lactose-free products, as there is no official “lactose-free” symbol in the national or European regulations. Each manufacturer can use its own logo on its product; therefore, this causes a great heterogeneity in the typology of symbols for the indication of a product that does not contain lactose.

The benefits of dairy in the diet at different stages of life have been widely recognized, and they are included in the dietary guidelines for the Spanish population, with a recommendation of two to three servings per day [27]. In fact, several recent studies show the importance of dairy products in our country, since they are consumed daily by the Spanish population [28], and they are the main source of calcium (53.1%) and phosphorous (26.1%), especially amongst younger population groups [29]. According to Olza et al., vitamin D contribution from dairy products is lower than that from fish and eggs amongst adults but is higher for children [30]. Moreover, research has shown that a high proportion of the Spanish population is not meeting the recommended intakes for calcium and vitamin D [29,30]. Consumption of yogurt and other fermented products is associated with improved health outcomes. Although dairy consumption is included in most dietary guidelines, there have been few specific recommendations for yogurt and fermented dairy products [31].

The compiled lactose-free FCDB included 327 lactose-free products, of which 123 were dairy lactose-free products, classified in 8 subgroups, the main ones being yogurt (*n* = 35, 25%), milk (*n* = 33, 24%), and cheese (*n* = 24, 17%). Data from DSM Food Specialties [32] showed that milk products are the main lactose-free product commercialized in Spain, and our data show only a small difference in availability. In addition, it must be noted that the yogurt and fermented products subgroup encompasses a greater variety in terms of fat levels, flavors, fruit, or sugar addition than the milk subgroup. Therefore, we can conclude that, nowadays, fourteen years after the first lactose-free milk appeared on the market, lactose-intolerant subjects have a complete and varied choice of lactose-free dairy products to include in their diets and comply with recommendations. This is a good example of how the food industry reformulates products according to consumer’s needs.

Regarding composition, our results revealed that butter and cream declared the highest energy contents, followed by cheese and ice cream, showing similar amounts of energy. Milk is the subgroup with the lowest energy content. Similarly, butter and cream had the highest contents of total fats and saturated fats. On the other hand, coffee, cocoa, tea, and infusions had the lowest fat content. 

Evaporated and condensed milks can be classified as food with a high carbohydrate content, in contrast to cheese. They also showed the highest sugar content, followed by ice cream and cheese. The cheese subgroup presented the lowest content of sugar and the highest protein content, in contrast to cream and butter, which had the lowest protein content. In addition, cheese presented the highest amount of salt, while other subgroups declared a low content of salt. Because matured cheeses contain a lower lactose content than fresh cheeses, matured cheeses may be consumed by lactose-intolerant people, even without a lactose-reducing process, thus allowing the consumption of nutritious dairy products in this population group [33].

As expected, when comparing the nutritional composition from lactose-free milk products against equivalent traditional milk (*n* = 18), lactose-free yogurts against equivalent traditional yogurts (*n* = 20), lactose-free cheeses against traditional lactose cheese (*n* = 30), lactose-free milkshakes and dairy beverages against traditional milkshakes and dairy beverages (*n* = 8), lactose-free butter and cream against traditional butter and cream (*n* = 8), and lactose-free dairy desserts against traditional dairy desserts (*n* = 8), we found no significant differences. Lactose-free products contained similar amounts of energy, fats, carbohydrates, sugars, fiber, protein, and salt as traditional ones. We did not find any similar studies in the literature conducting comparisons for lactose-free products; however, Calvo-Lerma et al. did find, in a sample of gluten-free products commercialized in Spain, that regular flour, bread, pasta and pizza had higher protein contents than their gluten-free equivalents. Similarly, gluten-free bread showed higher total and saturated fat quantities [34]. The fact that lactose elimination does not play a crucial role in the product’s sensory or technological properties determines that lactose-free products do not need to be reformulated as gluten-free products do, and, therefore, their nutrient contribution is equivalent to those of traditional products.

In fact, the only perceived differences between lactose-free dairy and traditional dairy are a sweeter taste and lower viscosity [35]. By adding lactase, the enzyme breaks lactose into glucose and galactose, thus enhancing sweetness, solubility, and flavor, as well as improving digestibility [12].

We need to stress that nutrient contents declared by labels are not always the result of chemical analysis but are calculated from food composition tables or FCBD based on their ingredients. Since this information is not provided by manufacturers, they might not represent the actual nutrient contribution accurately, as observed by other authors [36]. Furthermore, micronutrient contents declared on food labels are limited or non-existent, only appearing when there is vitamin or mineral addition in fortified products. 

Concerning added sugars that were specifically declared on the ingredient list of the labels (not dairy-intrinsic sugars), these were identified in 31% of products (n = 39), sucrose being the main one (82%), while LNCS were identified only in 9.6% (n = 12) of products. It is noteworthy that yogurt was the subgroup with a higher frequency of added sugars and LNCS. As current European labeling regulations do not require the manufacturer to declare the amount of added sugar in the nutrition label [8], we were not able to calculate the proportions of added sugars in comparison with intrinsic sugars from lactose-free products. In recently published research by Samaniego et al. [37], dairy products were one of the main groups where added sugars, as well as LNCS, were found on the Spanish market, so it is surprising that lactose-free products showed such a low frequency of LNCS addition.

Lactose-free products are fortified with vitamins A, D, E and B_9_, and calcium. Dairy food fortification with vitamin D is important to counteract dietary inadequacy in Europe, since there is a big gap between actual intakes (3–7 µg/day) and dietary reference intakes (10–20 µg/day) [38]. Many individuals with real or perceived lactose intolerance avoid dairy and ingest inadequate amounts of calcium and vitamin D, which may predispose them to decreased bone accrual, osteoporosis, and other adverse health outcomes [1]. Therefore, food fortification is rendered important to avoid inadequacies. In Spain, food fortification is not mandatory, but dairy is usually fortified with vitamins A, D, E, niacin, B_6_, C, and folate, as well as with calcium, iron, magnesium, and phosphorus [39]. In fact, only dairy foods are fortified with vitamin D, and the consumption of fortified dairy is increasing annually, topping almost 64 g per person per day [39]. In lactose-free products, we found that 24% of them were fortified. To prevent inadequate vitamin D and calcium intakes in lactose intolerants, more products need to be fortified. 

According to the NOVA classification, the database includes many ultra-processed products (55.5%), but this is due to a large number and variety of sweetened and flavored yogurts, among other minority dairy subgroups, and products with a dairy component. However, the other half of the database corresponds to Group 1, unprocessed or minimally processed foods (milk and natural unsweetened yogurts), and Group 3, processed foods (cheeses), these being the main lactose-free subgroups.

To our knowledge, this lactose-free FCDB is the first one to compile lactose-free branded products available on the Spanish market, and it represents a new resource for dietary assessment of the lactose-intolerant population group. It is also the first step and basis to monitor data on key nutrients like added sugars and sodium, which are of public health concern for their excessive intake in westernized diets. In accordance with the “collaboration plan for the improvement of the composition of food and beverages” conceived by the Spanish health authorities, the reformulation of specific food groups and subgroups has been encouraged amongst manufacturers to decrease added sugars, saturated fats and salt content, and dairy products are no exception [40].

## 5. Conclusions

According to our study, lactose-intolerant subjects have a complete and varied choice of lactose-free dairy products to include in their diets and comply with recommendations for dairy consumption. We have identified and described 327 lactose-free dairy products and products that contain dairy and dairy derivatives in their formulation. The lactose-free FCDB can be used as a helpful tool to assess the nutrient quality of these products, as well as to evaluate dietary intakes of lactose-intolerant individuals, and to monitor the evolution of these products’ formulation. Nutritional composition in terms of macronutrients and salt is not significantly different from lactose-containing products. Nonetheless, the frequency of vitamin D and calcium fortification is low. Moreover, we also identified an intriguing overuse of the lactose-free label.

Finally, processed product variety, rotation and availability are dynamic and rapidly changing, as new products are continuously reaching the market while others might be eliminated, making it challenging to maintain an updated FCDB [36]. In addition, manufacturers can change food formulations over periods of time, and online resources might become outdated, yet again underlining the need for frequent FCBD updating [36] and future research on food composition.

### 5.1. Strengths 

Lactose-free products are commonly not included in food composition databases. This is the first work that systematically compiled a statistically important number of this type of products (*n* = 327). In terms of data compilation and availability, online retailers’ platforms were a useful tool to identify and acquire a representative number of products. Products were compiled and organized using standardized classification systems (LanguaL™ Thesaurus EuroFIR [16] and FoodEx2 [17]), which are widely used in European FCDBs.

### 5.2. Limitations

A few limitations to our study should be noted. Firstly, we relied on data declared by manufacturers as we did not perform any chemical analysis of the proximate composition of products. Secondly, nutrient contents declared by labels are not always the result of chemical analysis but are calculated from FCDBs based on their ingredients. This information is not provided by manufacturers.

Finally, the food industry reformulates its products and launches new products continuously, challenging research and updates on the FCDBs to provide sound food composition data. 

## Figures and Tables

**Table 1:** Label-declared nutrient content of lactose-free dairy food subgroups per 100 g of edible portion.

	Energy (kcal)	Total Fats (g)	Saturated Fats (g)	Carbohydrates (g)	Sugars (g)	Protein (g)	Salt (g)
Whole Milk	47.27 ± 10.79	1.70 ± 1.24	1.13 ± 0.86	4.82 ± 0.28	4.68 ± 0.25	3.18 ± 0.23	0.10 ± 0.03
Semi-skimmed milk	45.66 ± 0.81	1.59 ± 0.02	1.05 ± 0.05	4.75 ± 0.15	4.75 ± 0.15	3.14 ± 0.08	0.12 ± 0.01
Skimmed milk	38.83 ± 7.76	0.63 ± 0.82	0.23 ± 0.13	4.88 ± 0.21	4.88 ± 0.21	3.48 ± 0.75	0.16 ± 0.05
Yogurt and fermented milk with sugar	93.20 ± 23.67	3.22 ± 2.35	2.05 ± 1.58	12.69 ± 2.74	10.93 ± 1.65	3.20 ± 0.56	0.12 ± 0.03
Yogurt and fermented milk (sugar-free)	45.31 ± 12.45	0.87 ± 1.39	0.54 ± 0.96	5.37 ± 1.93	5.08 ± 1.79	4.10 ± 0.84	0.13 ± 0.04
Dairy desserts	119.00 ± 20.11	7.73 ± 13.47	1.94 ± 0.57	19.49 ± 3.57	15.53 ± 3.65	3.20 ± 0.78	0.13 ± 0.04
Butter and cream	331.38 ± 189.09	34.00 ± 22.03	22.54 ± 15.03	4.44 ± 375	3.69 ± 3.97	1.91 ± 0.63	0.16 ± 0.13
White Cheese	215.33 ± 73.29	17.98 ± 8.40	12.54 ± 5.97	3.09 ± 0.99	2.63 ± 1.14	10.15 ± 4.46	1.09 ± 0.76
Processed cheese	328.55 ± 62.36	26.03 ± 5.84	16.88 ± 3.92	0.39 ± 0.57	0.36 ± 0.51	22.83 ± 4.69	1.75 ± 0.47
Milkshakes and dairy beverages	89.33 ± 96.14	1.02 ± 0.42	0.69 ± 0.29	13.33 ± 14.64	12.17 ± 11.77	6.13 ± 8.95	0.2 ± 0.24
Evaporated and condensed milk	226.33 ± 94.68	2.13 ± 3.34	1.23 ± 1.96	43.63 ± 29.73	43.63 ± 29.73	8.03 ± 1.50	0.24 ± 0.01
Ice-cream	283.33 ± 88.03	18.30 ± 9.02	12.76 ± 6.48	24.00 ± 0.00	22.33 ± 1.15	4.46 ± 1.26	0.11 ± 0.01
Chocolates	565.00 ± 14.67	36.55 ± 3.13	21.03 ± 2.16	51.08 ± 6.53	50.48 ± 6.53	7.00 ± 1.83	0.13 ± 0.03
Cold cuts and sausages	81.50 ± 0.00	1.00 ± 0.00	0.30 ± 0.00	3.50 ± 0.00	1.50 ± 0.00	14.50 ± 0.00	1.80 ± 0.00
Canned and precooked dishes	213.98 ± 17.56	7.93 ± 1.49	4.35 ± 1.04	25.87 ± 2.39	1.77 ± 0.19	9.17 ± 0.89	1.30 ± 0.13
Mixed refreshing drinks	27.20 ± 9.40	0.15 ± 0.10	0.00 ± 0.00	6.18 ± 2.30	5.44 ± 2.50	0.48 ± 0.10	0.16 ± 0.10

Results are shown as average ± standard deviation.

**Table 2:** Energy, macronutrients, and salt content (per 100 g) in lactose-free products and their lactose-containing counterparts, based on the label-declared nutritional information.

	Energy (kcal)		Total Fats (g)		Saturated Fats (g)		Carbohydrates (g)		Sugars (g)		Protein (g)		Salt (g)	
	Lactose Free	Traditional Products	Lactose Free	Traditional Products	Lactose Free	Traditional Products	Lactose Free	Traditional Products	Lactose Free	Traditional Products	Lactose Free	Traditional Products	Lactose Free	Traditional Products
Whole milk	47.2 ± 10.7	64.1 ± 1.9	1.7 ± 1.2	3.6 ± 0.0	1.1 ± 0.8	2.3 ± 0.1	4.8 ± 0.2	4.7 ± 0.1	4.8 ± 0.2	4.7 ± 0.1	3.1 ± 0.2	3.2 ± 0.3	0.1 ± 0.0	0.1 ± 0.0
Semi-skimmed milk	45.6 ± 0.8	45.6 ± 0.5	1.5 ± 0.0	1.5 ± 0.0	1.0 ± 0.0	1.0 ± 0.0	4.7 ± 0.1	4.7 ± 0.0	4.7 ± 0.1	4.7 ± 0.0	3.1 ± 0.0	3.1 ± 0.0	0.1 ± 0.0	0.1 ± 0.0
Skimmed milk	38.8 ± 7.7	39.1 ± 7.6	0.6 ± 0.8	0.7 ± 0.7	0.2 ± 0.1	0.2 ± 0.1	4.8 ± 0.2	4.9 ± 0.1	4.8 ± 0.2	4.9 ± 0.1	3.4 ± 0.7	3.3 ± 0.3	0.1 ± 0.0	0.1 ± 0.0
Yogurt and fermented milk with sugar	93.2 ± 23.6	83.7 ± 20.8	3.2 ± 2.3	2.3 ± 1.9	2.0 ± 1.5	1.6 ± 1.4	12.6 ± 2.7	12.2 ± 0.9	10.9 ± 1.6	11.7 ± 1.1	3.2 ± 0.5	2.9 ± 0.3	0.1 ± 0.0	0.1 ± 0.0
Yogurt and fermented milk (sugar-free)	45.3 ± 12.4	55.3 ± 17.0	0.8 ± 1.3	2.0 ± 2.4	0.5 ± 0.9	1.3 ± 1.8	5.3 ± 1.9	5.1 ± 1.1	5.0 ± 1.7	4.8 ± 1.2	4.1 ± 0.8	3.8 ± 0.6	0.1 ± 0.0	0.1 ± 0.0
Dairy desserts	119.0 ± 20.1	129.4 ± 15.1	7.7 ± 13.4	3.2 ± 0.8	1.9 ± 0.5	1.9 ± 0.6	19.4 ± 3.5	21.7 ± 3.1	15.5 ± 3.6	17.8 ± 2.9	3.2 ± 0.7	3.2 ± 0.7	0.1 ± 0.0	0.1 ± 0.0
Butter and cream	331.3 ± 189.0	377.0 ± 220.5	34.0 ± 22.0	39.3 ± 25.7	22.5 ± 15.0	26.3 ± 17.4	4.4 ± 3.7	4.0 ± 3.1	3.6 ± 3.9	3.2 ± 3.2	1.9 ± 0.6	1.6 ± 0.9	0.1 ± 0.1	0.1 ± 0.3
White cheese	215.3 ± 73.2	213.1 ± 55.4	17.9 ± 8.4	18.2 ± 7.3	12.5 ± 5.9	12.4 ± 5.2	3.0 ± 0.9	3.5 ± 0.7	2.6 ± 1.1	3.1 ± 0.5	10.1 ± 4.4	8.6 ± 3.3	1.0 ± 0.7	0.9 ± 0.1
Processed cheese	328.5 ± 62.3	338.3 ± 68.3	26.0 ± 5.8	26.9 ± 6.3	16.8 ± 3.9	8.1 ± 4.5	0.3 ± 0.5	1.7 ± 2.5	0.3 ± 0.5	1.3 ± 2.1	22.8 ± 4.6	22.1 ± 6.1	1.7 ± 0.4	1.7 ± 0.6
Milkshakes and dairy beverages	89.3 ± 96.1	56.8 ± 15.9	1.0 ± 0.4	1.1 ± 0.4	0.6 ± 0.2	0.7 ± 0.2	13.3 ± 14.6	8.6 ± 3.8	12.1 ± 11.7	8.5 ± 3.7	6.1 ± 8.9	2.8 ± 0.7	0.2 ± 0.2	0.1 ± 0.0

Results are shown as average ± standard deviation.

**Table 3:** Number of lactose-free dairy products with labelling claims.

Dairy Lactose-Free Products	*n*	% of Total
Nutritional claims	23	19
Health claims	2	2
Disease risk reduction claims	0	0
Total products with claims	25	21

**Table 4:** Type of added sugars (**A**) and low- or no-calorie sweeteners (LNCS) (**B**), present in lactose-free products ingredients lists.

(A) Type of Declared Added Sugars	*n*	% of Total
Sucrose	32	26
Sucrose + Glucose syrup	2	2
Sucrose + Glucose-Fructose syrup	2	2
Glucose-Fructose syrup	2	2
Sucrose, Caramel (Sucrose, Glucose syrup)	1	1
Total	39	33
**(B) Type of Declared LNCS**	***n***	**% of Total**
Acesulfame K + Sucralose	8	7
Sucralose	3	1
Steviol glycosides + Acesulfame K + Sucralose	1	2
Total	12	10

**Table 5:** Proportion of fortified products amongst lactose-free dairy products.

Lactose-Free Food Dairy Subgroups	Number of Fortified Products	% of Total
Milk	21	17
Yogurt and fermented milks	0	0
Cheese	0	0
Milkshakes and dairy beverages	3	2
Dairy desserts	0	0
Butter and cream	0	0
Evaporated and condensed milk	0	0
Ice-cream	0	0
Total fortified products	24	19

**Table 6:** Use of lactase as an ingredient in lactose-free dairy products.

Lactose-Free Food Dairy Subgroups	Number of Products Containing Lactase	% of Total
Milk	28	23
Yogurt and fermented milks	32	26
Cheese	10	8
Milkshakes and dairy beverages	5	4
Dairy desserts	3	2
Butter and cream	1	1
Evaporated and condensed milk	3	2
Ice cream	0	0
Total products containing lactase	82	66

## Data Availability

The data presented in this study are available on request from the corresponding author.
